# A manually curated compendium of expression profiles for the microbial cell factory *Corynebacterium glutamicum*

**DOI:** 10.1038/s41597-022-01706-7

**Published:** 2022-10-01

**Authors:** Angela Kranz, Tino Polen, Christian Kotulla, Annette Arndt, Graziella Bosco, Michael Bussmann, Ava Chattopadhyay, Annette Cramer, Cedric-Farhad Davoudi, Ursula Degner, Ramon Diesveld, Raphael Freiherr von Boeselager, Kim Gärtner, Cornelia Gätgens, Tobias Georgi, Christian Geraths, Sabine Haas, Antonia Heyer, Max Hünnefeld, Takeru Ishige, Armin Kabus, Nicolai Kallscheuer, Larissa Kever, Simon Klaffl, Britta Kleine, Martina Kočan, Abigail Koch-Koerfges, Kim J. Kraxner, Andreas Krug, Aileen Krüger, Andreas Küberl, Mohamed Labib, Christian Lange, Christina Mack, Tomoya Maeda, Regina Mahr, Stephan Majda, Andrea Michel, Xenia Morosov, Olga Müller, Arun M. Nanda, Jens Nickel, Jennifer Pahlke, Eugen Pfeifer, Laura Platzen, Paul Ramp, Doris Rittmann, Steffen Schaffer, Sandra Scheele, Stephanie Spelberg, Julia Schulte, Jens-Eric Schweitzer, Georg Sindelar, Ulrike Sorger-Herrmann, Markus Spelberg, Corinna Stansen, Apilaasha Tharmasothirajan, Jan van Ooyen, Philana van Summeren-Wesenhagen, Michael Vogt, Sabrina Witthoff, Lingfeng Zhu, Bernhard J. Eikmanns, Marco Oldiges, Georg Schaumann, Meike Baumgart, Melanie Brocker, Lothar Eggeling, Roland Freudl, Julia Frunzke, Jan Marienhagen, Volker F. Wendisch, Michael Bott

**Affiliations:** 1 IBG-1: Biotechnology, Institute of Bio- and Geosciences, Forschungszentrum Jülich, D-52425 Jülich, Germany; 2 IBG-4: Bioinformatics, Institute of Bio- and Geosciences, Forschungszentrum Jülich, D-52425 Jülich, Germany; 3 Institute of Microbiology and Biotechnology, University of Ulm, D-89069 Ulm, Germany; 4 SenseUp GmbH, c/o Campus Forschungszentrum, Wilhelm-Johnen-Strasse, D-52425 Jülich, Germany; 5 Genetics of Prokaryotes, Biology & CeBiTec, Bielefeld University, Universitaetsstr. 25, D-33615 Bielefeld, Germany

**Keywords:** Metabolic engineering, Bacterial genetics

## Abstract

*Corynebacterium glutamicum* is the major host for the industrial production of amino acids and has become one of the best studied model organisms in microbial biotechnology. Rational strain construction has led to an improvement of producer strains and to a variety of novel producer strains with a broad substrate and product spectrum. A key factor for the success of these approaches is detailed knowledge of transcriptional regulation in *C. glutamicum*. Here, we present a large compendium of 927 manually curated microarray-based transcriptional profiles for wild-type and engineered strains detecting genome-wide expression changes of the 3,047 annotated genes in response to various environmental conditions or in response to genetic modifications. The replicates within the 927 experiments were combined to 304 microarray sets ordered into six categories that were used for differential gene expression analysis. Hierarchical clustering confirmed that no outliers were present in the sets. The compendium provides a valuable resource for future fundamental and applied research with *C. glutamicum* and contributes to a systemic understanding of this microbial cell factory.

**Table:** 

Measurement(s)	Gene Expression Analysis
Technology Type(s)	Two Color Microarray
Factor Type(s)	WT condition A vs. WT condition B • Plasmid-based gene overexpression in parental strain vs. parental strain with empty vector control • Deletion mutant vs. parental strain
Sample Characteristic - Organism	Corynebacterium glutamicum
Sample Characteristic - Environment	laboratory environment
Sample Characteristic - Location	Germany

## Background & Summary

*Corynebacterium glutamicum* is a Gram-positive, facultatively anaerobic soil bacterium, which was discovered in the 1950s as a natural L-glutamate producer^1^. Nowadays, *C. glutamicum* is established as an important industrial microorganism used for the large-scale production of L-glutamate (3.2 million tons/year) and L-lysine (2.6 million tons/year)^2–6^. Efficient strains for the synthesis of several other amino acids such as L-leucine^7^, L-isoleucine^8^, L-valine^9–12^, L-arginine^13^, or L-histidine^14,15^ have also been constructed. After the availability of the genome sequence^16,17^, the product spectrum accessible with *C. glutamicum* was continuously extended and now includes for example various organic acids^18–20^, biofuels such as ethanol^21^, isobutanol^22,23^ or 2-methyl-1-butanol and 3-methyl-1-butanol^24^, carotenoids^25^, plant secondary metabolites such a plant polyphenols^26–28^, and heterologous proteins^29^ such as antibodies. An essential prerequisite for the development of production strains of *C. glutamicum* by re-routing cellular metabolism was the development of efficient and reliable genetic tools enabling for example the deletion and overexpression of genes, genomic integration, or the introduction of modifications in promoter and operator (i.e. regulator-binding) sequences^30–34^.

Rational development of microbial production strains does not only require knowledge about the metabolic network^35^, but also of the regulatory networks that control metabolic fluxes. To analyse transcriptional changes of gene expression in response to environmental changes and metabolic shifts in the wild-type as well as engineered strains on a genome-wide scale, transcriptomics methods such as DNA microarrays or RNA-Seq are used. For *C. glutamicum*, many studies on transcriptional regulators (TRs)^36–38^, including two-component signal transduction systems^39^ and σ factors^40^, have been performed to elucidate the complex network of transcriptional regulation in this bacterium. To understand the physiological functions of individual TRs, their target genes (also called regulon) need to be identified, e.g. by comparing changes in gene expression in strains lacking or overexpressing individual TR genes. In order to distinguish direct and indirect influences on target gene expression, the binding of the TRs to promoters has to be analyzed. For example, *in vivo* DNA-binding sites of individual TRs can be identified by chromatin immunoprecipitation (ChIP) in combination with microarrays (ChIP-Chip) or high-throughput sequencing (ChIP-Seq)^41^, or by using chromatin affinity purification followed by sequencing (ChAP-Seq)^42^.

Of the 159 genes encoding either DNA-binding TRs (139), response regulators of two-component systems (13), or σ factor subunits of the RNA polymerase (seven) in the genome of *C. glutamicum*^17,38^, 93 TRs^36,37,43^, seven two-component systems^39,44–47^, and all seven sigma factors^40,48–50^ have been characterized so far. Information about the respective regulatory functions can be found in the web-based analysis platform CoryneRegNet^43,51^. Despite this huge progress since the availability of the *C. glutamicum* genome sequences in 2003^16,17^, the understanding of the transcriptional regulatory network in this bacterium is still far from being complete. In general, the possible number of regulatory interactions is equal to the number of transcription factors multiplied by the total number of genes in the genome multiplied by the number of environmental contexts in which the cell might find itself^52^. Therefore, comprehensive analysis of gene expression changes measured in a broad variety of different strains and growth conditions is necessary by using, for example, methods such as data mining or strategies such as knowledge discovery in databases^53–55^.

Over the past 20 years, researchers of the Institute of Bio- and Geosciences 1: Biotechnology (IBG-1, until 2010 named Institute of Biotechnology 1) at Forschungszentrum Jülich (Germany) have performed and collected the results of 1,146 microarray experiments in an in-house database^56^. The majority of these microarray data (671 experiments) is not yet available publicly. Thus, in this study, we re-evaluated all experiments stored in our in-house database according to the Minimum Information About Microarray Experiments (MIAME) standards^57^, curated them manually and uploaded them as a superseries of 927 experiments to Gene Expression Omnibus (GEO). Furthermore, we categorized the experiments and combined the replicates to 304 microarray sets that were used for differential gene expression analysis and hierarchical clustering analysis. This approach follows the FAIR principles^58,59^ and allows other researchers to use our data for future studies and contribute to a systemic understanding of the transcriptional regulatory network and the biology of *C. glutamicum* and its biotechnological application. Furthermore, the resulting knowledge gained for *C. glutamicum* can also benefit the understanding of phylogenetically related Actinobacteria, for example mycobacteria^60,61^.

**Fig. 1:**
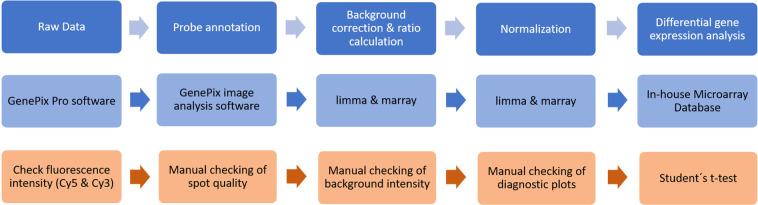
Processing workflow applied to Agilent, Operon and PCR product-based microarrays. Boxes in blue represent the performed steps, boxes in light blue show the programs that were used, and the boxes in orange contain the quality checking procedures that were applied during the respective steps.

**Fig. 2:**
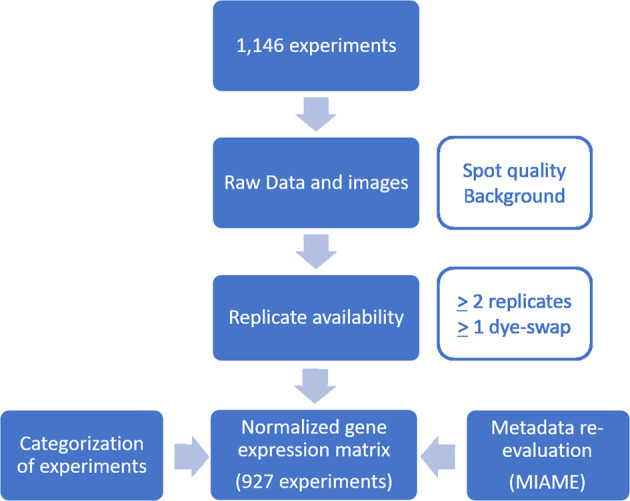
DNA microarray re-evaluation pipeline. Overall, 1,146 experiments were re-evaluated during this study. After the re-evaluation and manual quality check, 927 high-quality experiments remained and were uploaded to GEO.

The complete workflow including processing of microarray experiments, re-evaluation of the data, and categorization is described in detail in the methods as well as validation section and visualized in Figs. 1 and 2.

## Methods

### Bacterial strains, plasmids, and cultivation conditions

All bacterial strains and plasmids that were used for microarray comparisons are listed in Supplementary Table S1. Strains bearing deletions, genomic integrations and/or promoter exchanges were constructed using a two-step homologous recombination protocol based on the suicide plasmid pK19mobsacB^62^. Construction of plasmids for (over-)expression of homologous or heterologous genes was performed by PCR amplification of the target sequences and cloning into suitable vectors by restriction and ligation or Gibson assembly^63^. The deletion of genes was confirmed by PCR. Aerobic cultivation of *C. glutamicum* strains under standard conditions was performed in 50 mL of lysogeny broth (LB)^64^, 50 mL brain-heart-infusion (BHI) medium, or 50 mL CGXII minimal medium^65^ in 500 mL shaking flasks at 30 °C. In many cases, 2% (w/v) or 4% (w/v) glucose was used as carbon source. Changes in the composition of the used CGXII medium, such as the use of different carbon sources or the addition of special chemicals, as well as changes in the cultivation temperature are indicated. Plasmids were introduced into the indicated strains usually by electroporation^66^. When required, gene expression was induced, e.g. by addition of isopropyl-β-D-thiogalactopyranoside (IPTG) to a final concentration between 10 µM and 1 mM as indicated or by addition of anhydrotetracycline (typically 250 ng/ml). Antibiotics were used at the following concentrations: kanamycin, 25 µg ml^−1^; chloramphenicol, 10 µg ml^−1^; spectinomycin, 100 µg ml^−1^. Cells were usually grown until mid-exponential phase (optical density at 600 nm (OD_600_) of ca. 4–5), harvested on ice, frozen in liquid nitrogen, and stored at −80 °C until further use for RNA isolation. In some experiments, cells were grown to an OD_600_ of ca. 5, washed in PBS buffer (137 m*M* NaCl, 2.7 m*M* KCl, 1.5 m*M* KH_2_PO_4_, 8 m*M* Na_2_HPO_4_, pH 7.4) or 0.9% (w/v) saline, transferred into new medium and harvested after cultivation for the indicated time. Experiments that required longer cultivation of the cells are designated accordingly. Aerobic and anaerobic cultivations in bioreactors were performed as described^67–69^. For every gene expression comparison, two to nine biological replicates were performed.

### RNA isolation and microarray hybridization

Total RNA was isolated using the RNeasy Kit (Qiagen, Hilden, Germany) or as described^70^. Equal amounts of RNA (15–30 µg) were used for random hexamer-primed synthesis of fluorescently labelled cDNA using the nucleotide analogues Cy3-dUTP or Cy5-dUTP (GE Healthcare, Eindhoven, Netherlands). Gene expression changes of two samples were compared using either self-made PCR product-based DNA microarrays^71^, custom-made DNA microarrays with 70-mer oligonucleotides obtained from Operon Biotechnologies (Cologne, Germany), or custom-made 4x44K 60mer DNA microarrays from Agilent Technologies (Waldbronn, Germany). Hybridization of mixtures of Cy3- and Cy5-labelled cDNA on the arrays and washing of the arrays were performed as described^7,72,73^. Routinely, at least one dye-swap experiment was performed per gene expression comparison to avoid batch effects by consistently labelling identical experimental groups with the same dye. All oligonucleotides spotted on the arrays are based on the genome sequence entry NC_006958^17^.

### Fluorescence determination and probe annotation

The fluorescence of DNA microarrays was determined at 532 nm (Cy3-dUTP) and 635 nm (Cy5-dUTP) at 5 or 10 µm resolution with a GenePix 4000B laser scanner and GenePix Pro Software (Molecular Devices, Sunnyvale, USA). Raw data files of fluorescence images were saved in TIFF format and further processed using the GenePix image analysis software. Probe annotation was used to map each spot on the scanned microarray chip to genes. For this purpose, gene array list (GAL) files designed specifically for the platform used were loaded into the analysis software and aligned to the probes to perform spot detection. Multiple probes that mapped onto the same gene ID were summarized by their median values. All GAL files were uploaded to the GEO database (GPL29897).

### Background correction, ratio calculation and normalization

The raw results were saved as GPR files and further processed using the BioConducter R packages limma and marray (http://www.bioconductor.org). To achieve background correction the respective limma function was used to subtract the background intensity from the foreground intensity for each spot. Afterwards, the Cy5/Cy3 or Cy3/Cy5 (for dye-swap experiments) ratios for each background-subtracted spot were calculated and normalized. The purpose of normalization is to remove sources of systemic variation in the measured fluorescence intensities that might influence the differential expression analysis (e.g. different labelling efficiencies, different scanning parameters or scanning properties). Here, we used the R package marray to perform loess normalization followed by diagnostic-plot generation (volcano plots, boxplots, MA-plots). Normalized ratios of medians reflecting the relative mRNA level were filtered for a signal-to-noise ratio ((F635Median/B635Median) or (F532Median/B532Median)) higher than 3.

### Differential gene expression analysis

For further analysis, loess-normalized data were stored in the in-house DNA microarray database^56^ including metadata about each experiment. The in-house DNA microarray database is a custom-made database based on mySQL running on a Linux host combined with a graphical user interface (self-made Java applications) allowing display and analysis of stored microarray data^56^. Routinely, replicates were compared with each other and obvious outlier experiments were removed. Afterwards, replicates belonging to the same experimental setup were combined to sets and then used to perform differential gene expression analysis. For this purpose, normalized log-transformed RNA levels from two or more biological replicate experiments (in case of dye-swaps the reciprocal values were used) were averaged and p-value calculation based on Student´s t-test was performed. For each set, only genes showing statistically significant expression ratios (*p*-value ≤ 0.05) were considered for further analysis steps. Overall, 927 individual microarray experiments were combined to 304 sets.

### Quality check

Experimental setup, sample preparation, microarray platform, and equipment used for scanning and analysis of the DNA microarrays can influence technical variation. This so-called batch effect can lead to incorrect results and subsequently to misinterpretation of results. To evaluate possible batch effects, we performed hierarchical clustering analysis using R. All results were visualized using the ggplot2 package in R.

## Data Records

The raw data (GPR files) for all 927 experiments alongside relevant metadata and unfiltered differential expression results (normalized ratio data) as a matrix were uploaded to the GEO database^74^.

In addition, the following files are freely available on Zenodo (10.5281/zenodo.6842664)^75^:

- Normalized expression matrix: Normalized signal intensities (for channel Cy5 and Cy3) resulting from the pre-processing of microarrays with the R packages limma and marray for every experiment.
- Filtered differential expression results: Matrix with significantly expressed genes (≥2-fold up- and ≥2-fold-down-regulated) with an adjusted *p*-value ≤ 0.05 for each set.

## Technical Validation

### Re-evaluation and categorization of microarray experiments

All downstream analysis steps of expression data rely on the quality of the data. Therefore, we manually re-evaluated and curated all microarray experiments (1,146) that were pre-processed as described in the Methods section and saved in our in-house microarray database before they were uploaded to the GEO database. The following criteria were applied for this purpose (Fig. 2):

- Two or more replicates were available for each experimental setup.
- At least one replicate for each experimental setup was a dye-swap experiment.
- Non-normalized raw data and images were available.
- Every image obtained (see Microarray data processing) was checked manually for overall quality regarding spot quantity and background fluorescence. Only those chips showing a spot coverage of at least 80% and only minor background were included in the next steps.
- Only experiments with metadata that fulfilled the MIAME criteria^57^ were considered for upload.

After re-evaluation, 927 experiments were uploaded to GEO.

**Fig. 3:**
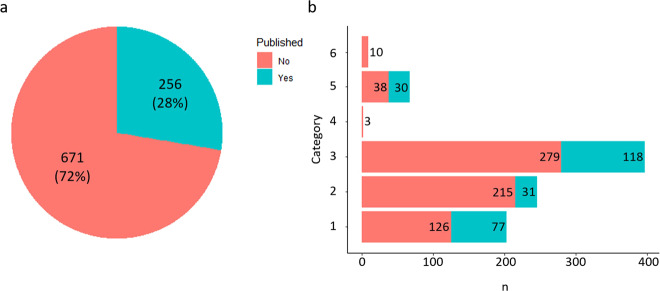
Overview about publication status and categorization of experiments. (**a**) Of the 927 experiments that are described in this publication, 256 had already been uploaded to GEO and/or are part of a publication in a peer-reviewed scientific journal. (**b**) Number of experiments in the six experimental categories (see section Re-evaluation and categorization of microarray experiments for further information) and publication status. Categories: (1) WT condition A *vs*. WT condition B. (2) Plasmid-based gene overexpression in parental strain *vs*. parental strain with empty vector control. (3) Deletion mutant *vs*. parental strain. (4) Gene silencing strain *vs*. parental strain. (5) Promoter exchange strain *vs*. parental strain. (6) Producer strain *vs*. WT or other producer strain.

Alongside the raw data uploaded to the GEO repository, the following detailed metadata was added: (i) Strain background (for further information about strains and plasmids used in this study see Supplementary Table S1). (ii) Media and other supplements used for cultivation of strains (see Methods for media composition). Usually, cells were harvested during the exponential growth phase (OD_600_ ca. 5) and then used for RNA isolation. Exceptions are stated. (iii) Number of replicates. (iv) The date (month and year) the experiment was performed. (v) The platform that was used for the specific microarray experiment (see Methods for further information). (vi) Additional information on the strains or genes that were deleted/over-expressed with respect to their function. (vii) If the strain/gene was already characterized and published, the associated PubMed ID (PMID) was added. (viii) If the microarray data were already part of a publication, the PMID and (if available) the GEO accession number were added. In order to ensure that the collected metadata are consistent and standardized, we used unified vocabularies to describe the experimental data. Of the 927 experiments 256 (28%) were already part of a publication (120) and/or uploaded to GEO (136) (Fig. 3a).

To facilitate the finding of specific data sets within this collection (Supplementary Table S2) by the user, they were categorized according to specific criteria: (1) WT condition A *vs*. WT condition B: experiments in which the transcriptome of *C. glutamicum* wild type (WT) was compared under two different growth conditions A and B. (2) Plasmid-based gene overexpression in parental strain *vs*. parental strain with empty vector control: experiments in which the influence of plasmid-based overexpression of homologous or heterologous genes in *C. glutamicum* parental strains (WT, deletion mutants or strains with genomically integrated promoter-fusions to genes encoding fluorescence proteins) on the transcriptome was studied by comparison with the parental strain carrying the plasmid vector only. (3) Deletion mutant *vs*. parental strain: experiments in which the transcriptome of a deletion mutant, i.e. a strain lacking one or several genes in the genome, that can also bear additional changes such as promoter exchanges, was compared with the transcriptome of the parental strain. (4) Gene silencing strain vs. parental strain: experiments in which the transcriptome of a strain with a silenced gene was compared with the transcriptome of the parental strain. (5) Promoter exchange strain *vs*. parental strain: experiments in which the transcriptome of strains bearing one or more genomically integrated promoter exchanges was compared with the transcriptome of the parental strain. (6) Producer strain *vs*. WT or other producer strain: experiments in which the transcriptome of a genetically engineered producer strain was compared with the transcriptome of the WT or another producer strain. An overview about the number of experiments that were associated with the described categories as well as their publication status can be found in Fig. 3b.

### Overview about microarray sets and experiments with respect to regulated genes and functional categorization

**Fig. 4:**
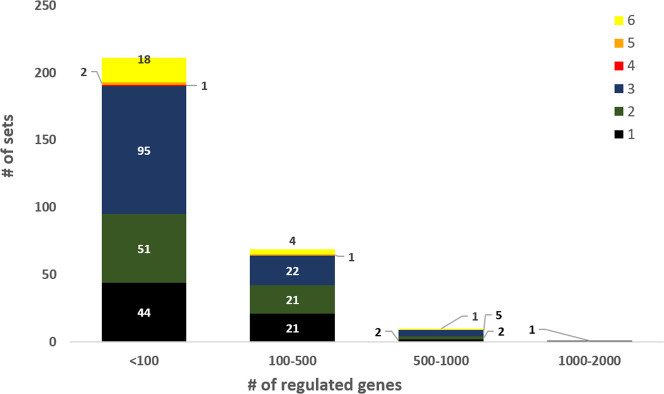
Number of sets with <100, 100–500, 500–1000, 1000–2000 and >2000 regulated genes (>2-fold up- and >2-fold-down-regulated, *p*-value ≤ 0.05) with respect to their categorization (see section Re-evaluation and categorization of microarray experiments for further information). Categories: (1) WT condition A *vs*. WT condition B. (2) Plasmid-based gene overexpression in parental strain *vs*. parental strain with empty vector control. (3) Deletion mutant *vs*. parental strain. (4) Gene silencing strain *vs*. parental strain. (5) Promoter exchange strain *vs*. parental strain. (6) Producer strain *vs*. WT or other producer strain.

Differentially regulated genes that are at least 2-fold up- or down-regulated (*p*-value ≤ 0.05) within microarray sets are of particular interest for scientific analysis to identify, for example, the regulon of a transcription factor or regulatory networks. Among the 304 sets of microarray comparisons described here, differentially regulated genes (≥2-fold) were found for 291 sets. The large majority of these sets (280) comprised less than 500 regulated genes out of 3,047 annotated genes, whereas 10 sets comprised between 500 and 1,000 regulated genes and one set showed an even larger number (>1,000) of regulated genes (Fig. 4). The latter two groups of sets and those without more than 2-fold regulated genes are listed in Supplementary Table S3. The trend that more than 90% of the sets contained less than 500 regulated genes is also reflected within five of the six categories described above.

**Fig. 5:**
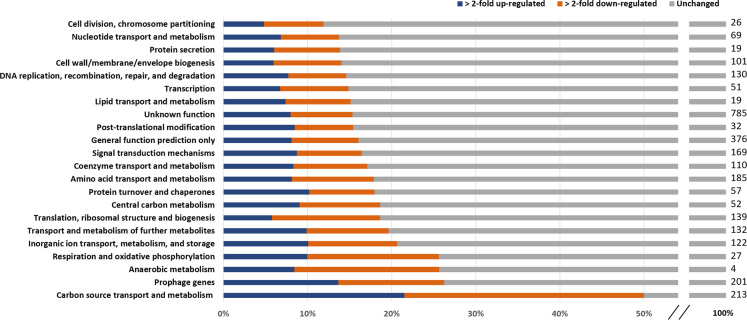
Proportion of genes associated to the functional categories^76^ that are ≥2-fold up-regulated (blue), ≥2-fold down-regulated (orange) or unchanged (grey) among the microarray sets described. The numbers at the right side indicate the genes allocated to the corresponding functional category.

In a previous study, the *C. glutamicum* genes were sorted into 22 functional groups^76^. An analysis of the percentage of regulated genes in these groups based on our microarray data revealed that between 12% and 50% were differentially regulated (Fig. 5). When comparing ≥2-fold up-regulated genes (5%–21%) with ≥2-fold down-regulated genes (7%–29%), it becomes obvious that in all 22 functional groups the number of both kinds of differentially regulated genes is very similar. With 50% the functional group “Carbon source transport and metabolism”^77^ has by far the highest proportion of differentially regulated genes. The smallest fraction of differentially regulated genes (12%) was found in the functional group “Cell division, chromosome partitioning”.

When analyzing the allocation to the 22 functional groups of those genes that were at least 30-times among the top 20 of regulated genes within all sets, genes involved in transport of carbon, inorganic ions and further metabolites were comparatively often differentially regulated (Supplementary Table S4, Supplementary Table S5).

**Fig. 6:**
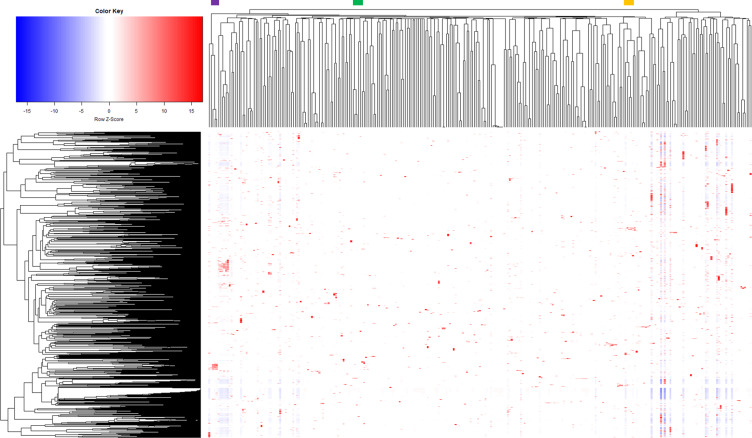
Hierarchical clustering of differentially regulated genes across sets. Unsupervised hierarchical clustering was performed using median log2 values of the 3021 genes that were differentially expressed (*p*-value ≤ 0.05) at least 2-fold within strain comparison, with uncentered Pearson correlations and complete clustering. Example clusters are highlighted with different coloured bars on top and are explained in the text.

To analyse the overall correlation between the sets, we performed hierarchical clustering of differentially regulated genes across all sets (Fig. 6). Overall, this analysis showed that several clusters could be identified and no obvious outlier sets were observed. In order to check the reliability of the analysis, we took a closer look at selected clusters with respect to their biological context. One cluster (purple bar) comprised experiments analysing the effects of protein secretion with different signal peptides and target proteins, which revealed differential expression of genes related to cell envelope stress^78^. A second cluster (green bar) involved experiments studying the influence of the DNA-intercalating agent mitomycin C, causing differential expression of a large set of stress response genes^77^. A third cluster (orange) comprised experiments analysing the effects of varying iron concentrations, in which the genes of the DtxR and RipA regulons were identified^78^. In summary, the correlation analysis showed no obvious batch effects, because many clusters comprised sets involving the usage of microarray chips from different platforms for the hybridization, the respective experiments were performed at different time points during the data collection period and/or the biological context favoured this cluster (Supplementary Table S6).

## Usage Notes

Here, we provide the first manually curated compendium of expression profiles for the microbial cell factory *C. glutamicum* comprising 927 high-quality microarray experiments that were summarized in 304 sets comprising the mean values for at least two replicates per experimental setup.

The availability of data for different strains as well as conditions and the resulting high variability among the amount of differentially regulated genes offers a valuable and rich resource for further analysis. For instance, it can be used to perform gene co-expression analysis to predict gene functions that are so far unknown^79^. Furthermore, our data collection can easily be integrated with other transcriptomic data (e.g. RNA-Seq or ChAP-Seq) to systematically identify transcriptional regulatory interactions by applying for example machine learning methods^53–55,80^. In addition, the data can also be used to study the perturbome of *C. glutamicum* to identify a set of core genes that are responsible for a common stress response towards different kind of perturbations^81^.

In summary, our manually curated compendium of expression profiles will have a high value for the community by contributing to the further systemic understanding and biotechnological exploitation of *C. glutamicum*.

## Supplementary information

Supplementary Table S1

Supplementary Table S2

Supplementary Table S3

Supplementary Table S4

Supplementary Table S5

Supplementary Table S6

## Data Availability

Raw data files of fluorescence images were analyzed by quantitative Image analysis using the GenePix image analysis software (GenePix Pro 6.0; https://axon-genepix-pro.software.informer.com/6.0/). In order to assign fluorescence signals to annotated genes gene array list (GAL) files were used (https://www.ncbi.nlm.nih.gov/geo/query/acc.cgi?acc=GSE169361)^74^. Processing of the raw data obtained (GPR-files) was performed with the BioConductor R-packages limma version 3.14 (https://bioconductor.org/packages/release/bioc/html/limma.html) and marray version 3.14 (https://bioconductor.org/packages/release/bioc/html/marray.html) to achieve background correction of spot intensities, ratio calculation/normalization, and diagnostic-plot generation for array quality control. Statistical analysis steps were performed within our in-house microarray database as well as with Excel (Microsoft).
