# Supplementary material for: A manually curated compendium of expression profiles for the microbial cell factory Corynebacterium glutamicum
Source: Sci Data. 2022 Oct 1;9:594. doi: 10.1038/s41597-022-01706-7 (PMC9526701; doi:10.1038/s41597-022-01706-7)
Supplement: Supplementary file 1 — Supplementary Table S1 [file 41597_2022_1706_MOESM1_ESM.docx]

**Supplementary Table S1.** *C. glutamicum* strains and plasmids used in this study. Km^R^, kanamycin resistance; Spc^R^, spectinomycin resistance; Cm^R^, chloramphenicol resistance.

| ***C. glutamicum***  **strain or plasmid** | **Description** | **Reference or source** |
| --- | --- | --- |
| **Strains** |  |  |
| ATCC13032 | Wild type (WT), biotin auxotrophic | ^1,2^ |
| Pcg1974-*eyfp* | ATCC 13032 derivative with the *eyfp* gene fused to the promoter of cg1974 from *C. glutamicum* | This study |
| ∆*citAB* | ATCC 13032 derivative with in-frame deletion of the *citAB* genes (cg0089-cg0090) | ^3^ |
| ∆*ilvA* ∆*panBC* | ATCC 13032 derivative with in-frame deletion of the *ilvA* (cg2334) and *panBC* genes (cg0149-cg0148) | ^4^ |
| ∆*iolR* | ATCC 13032 derivative with in-frame deletion of the *iolR* gene (cg0196) | ^5^ |
| ∆cg0210 | ATCC 13032 derivative with in-frame deletion of the cg0210 gene | This study |
| ∆*phdR* | ATCC 13032 derivative with in-frame deletion of the *phdR* gene (cg0343) | ^6^ |
| ∆*cyaB* | ATCC 13032 derivative with in-frame deletion of the *cyaB* gene (cg0375) | ^7^ |
| ∆*ramB* ∆*sdhCAB* | ATCC 13032 derivative with in-frame deletion of the *ramB* (cg0444) and *sdhCAB* genes (cg0445-cg0447) | ^8^ |
| ∆*csoR* | ATCC 13032 derivative with in-frame deletion of the *csoR* gene (cg0463) | This study |
| ∆*esrl* | ATCC 13032 derivative with in-frame deletion of the *esrl* gene (cg0706) | ^9^ |
| ∆*esrSR* | ATCC 13032 derivative with in-frame deletion of the *esrSR* genes (cg0707-cg0709, originally named *cgtS7* and *cgtR7*) | ^10,9^ |
| ∆*mntR* | ATCC 13032 derivative with in-frame deletion of the *mntR* gene (cg0741) | ^11^ |
| ∆cg0764 | ATCC 13032 derivative with in-frame deletion of the cg0764 gene | This study |
| silenced*icpA* | ATCC 13032 derivative with the silenced *icpA* gene (cg0847) | This study |
| ∆*whiB* | ATCC 13032 derivative with in-frame deletion of the *whiB* gene (cg0850) | This study |
| ∆*mtrA* | ATCC 13032 derivative with in-frame deletion of the *mtrA* gene (cg0862) | ^12^ |
| ∆*mtrAB* | ATCC 13032 derivative with in-frame deletion of the *mtrAB* genes (cg0862-cg0864) | ^13^ |
| ∆*mtrB* | ATCC 13032 derivative with in-frame deletion of the *mtrB* gene (cg0864) | ^12^ |
| ∆*sigH* | ATCC 13032 derivative with in-frame deletion of the *sigH* gene (cg0876) | ^14^ |
| ∆*cgtSR2* | ATCC 13032 derivative with in-frame deletion of the *cgtSR2* genes (cg0997-cg0996) | This study |
| ∆*htrA* | ATCC 13032 derivative with in-frame deletion of the *htrA* gene (cg0998) | This study |
| ∆*cgtSR10* | ATCC 13032 derivative with in-frame deletion of *cgtSR10* genes (cg1083-cg1084) | This study and ^10^ |
| ∆*ripA* | ATCC 13032 derivative with in-frame deletion of the *ripA* gene (cg1120) | ^15^ |
| ∆*sigE* | ATCC 13032 derivative with in-frame deletion of the *sigE* gene (cg1271) | This study |
| ∆*cydAB* | ATCC 13032 derivative with in-frame deletion of the *cydAB* genes (cg1301-cg1300) | ^16^ |
| ∆*cydAB* ∆*qcrBAC* | ATCC 13032 derivative with in-frame deletion of the *cydAB* (cg1301) and *qcrBAC* genes (cg2403-cg2405) | This study |
| ∆*rosR* | ATCC 13032 derivative with in-frame deletion of the *rosR* gene (cg1324) | ^17^ |
| ∆*atpBEF* | ATCC 13032 derivative with in-frame deletion of the *atpBEF* genes (cg1362-cg1364) | This study |
| ∆*atpBEFHAGDC* | ATCC 13032 derivative with in-frame deletion of the *atpBEFHAGDC* genes (cg1362-cg1369) | ^18^ |
| ∆*odhI* | ATCC 13032 derivative with in-frame deletion of the *odhI* gene (cg1630) | This study |
| ∆*ftsR* | ATCC 13032 derivative with in-frame deletion of the *ftsR* gene (cg1631) | ^19^ |
| ∆cg1633 | ATCC 13032 derivative with in-frame deletion of the cg1633 gene | This study |
| ∆*ndh* | ATCC 13032 derivative with in-frame deletion of the *ndh* gene (cg1656) | ^20^ |
| ∆*ndh* ∆*mdh* | ATCC 13032 derivative with in-frame deletion of the *ndh* (cg1656) and *mdh* genes (cg2613) | ^20^ |
| ∆*pup* | ATCC 13032 derivative with in-frame deletion of the *pup* gene (cg1689) | ^21^  ^22^ |
| ∆*acn* | ATCC 13032 derivative with in-frame deletion of the *acn* gene (cg1737) | ^23^ |
| ∆*alpC* | ATCC 13032 derivative with in-frame deletion of the *alpC* gene (cg1892) | ^24^ |
| ∆CGP3 | ATCC 13032 derivative with in-frame deletion of the prophage CGP3 (cg1890-cg2071) genes | ^25^ |
| ∆*gntR1* | ATCC 13032 derivative with in-frame deletion of the *gntR1* gene (cg2783) | ^26^ |
| ∆*gntR2* | ATCC 13032 derivative with in-frame deletion of the *gntR2* gene (cg1935) | ^26^ |
| ∆*gntR1* ∆*gntR2* | ATCC 13032 derivative with in-frame deletion of the *gntR1* (cg2783) and *gntR2* genes (cg1935) | ^26^ |
| ∆*sigB* | ATCC 13032 derivative with in-frame deletion of the *sigB* gene (cg2102) | This study |
| ∆cg2040 | ATCC 13032 derivative with in-frame deletion of the cg2040 gene | This study |
| ∆*aceE* | ATCC 13032 derivative with in-frame deletion of the gene *aceE* (cg2466) | ^27^ |
| ∆*aceEureD-E188** | ATCC 13032 derivative with in-frame deletion of the *aceE* gene and non-sense mutation in *ureD* (exchange of GAG (Glu188) to stop codon) | ^28^ |
| ∆*aceE*∆*pyc* | ATCC 13032 derivative with in-frame deletion of the *aceE* (cg2466) and *pyc* (cg0791) genes | ^29^ |
| ∆*dtxR* | ATCC 13032 derivative with in-frame deletion of the *dtxR* gene (cg2103) | ^30^ |
| ∆*fruR* | ATCC 13032 derivative with in-frame deletion of the *fruR* gene (cg2118) | This study |
| ∆*recA* | ATCC 13032 derivative with in-frame deletion of the *recA* gene (cg2141) | ^31^ |
| ∆cg2151 | ATCC 13032 derivative with in-frame deletion of the cg2151 gene | This study |
| ∆*clgR* | ATCC 13032 derivative with in-frame deletion of the *clgR* gene (cg2152) | ^14^ |
| ∆*chrSA* | ATCC 13032 derivative with in-frame deletion of the *chrSA* genes (cg2201-cg2200, initially named *cgtS8* and *cgtR8*) | ^10,32^ |
| ∆*chrSA* ∆*hrrSA* | ATCC 13032 derivative with in-frame deletion of the *chrSA* (cg2201-cg2200) and *hrrSA* genes (cg3248-cg3247) | This study |
| ∆*pcrCAB* | ATCC 13032 derivative with in-frame deletion of the *pcrCAB* genes (cg2403-cg2405) | This study |
| ∆*lldR* | ATCC 13032 derivative with in-frame deletion of the *lldR* gene (cg3224) | ^33^ |
| ∆*hrrSA* | ATCC 13032 derivative with in-frame deletion of the *hrrSA* genes (cg3248-cg3247) | ^10,34^ |
| ∆*hrrA* | ATCC 13032 derivative with in-frame deletion of the *hrrA* gene (cg3247) | ^34^ |
| ∆*qcrA* | ATCC 13032 derivative with in-frame deletion of the *qcrA* gene (cg2404) | ^35^ |
| ∆*surf1* | ATCC 13032 derivative with in-frame deletion of the *surf1* gene (cg2460) | ^36^ |
| ∆*ctiP* | ATCC 13032 derivative with in-frame deletion of the *ctiP* gene (cg2699) | ^37^ |
| ∆*phoDBC* | ATCC 13032 derivative with in-frame deletion of the *phoD* (cg2485), *phoB* (cg2700) and *phoC* (cg3393) genes | This study |
| ∆*fasR* | ATCC 13032 derivative with in-frame deletion of the *fasR* gene (cg2737) | ^38^ |
| ∆cg2750 | ATCC 13032 derivative with in-frame deletion of the cg2750 gene | ^37^ |
| ∆*cpdA* | ATCC 13032 derivative with in-frame deletion of the *cpdA* gene (cg2761) | ^39^ |
| ∆*ctaD* | ATCC 13032 derivative with in-frame deletion of the *ctaD* gene (cg2780) | ^37^ |
| ∆cg2784 | ATCC 13032 derivative with in-frame deletion of the cg2784 gene | This study |
| ∆*ramA* | ATCC 13032 derivative with in-frame deletion of the *ramA* gene (cg2831) | ^40^ |
| ∆cg2842 | ATCC 13032 derivative with in-frame deletion of the cg2842 gene | This study |
| ∆*ipsA* | ATCC 13032 derivative with in-frame deletion of the *ipsA* gene (cg2910) | ^41^ |
| ∆*cgtSR5* | ATCC 13032 derivative with in-frame deletion of the *cgtSR5* genes (cg2948-cg2947) | This study |
| ∆*copRS* | ATCC 13032 derivative with in-frame deletion of the *copRS* genes (cg3285-cg3284) | ^10,42^ |
| *∆copRS ∆cgtSR5* | ATCC 13032 derivative with in-frame deletion of the *copRS* (cg3285-cg3284) and *cgtSR5* genes (cg2948-cg2947) | This study |
| ∆*glnX* | ATCC 13032 derivative with in-frame deletion of the *glnX* gene (cg3044) | This study |
| ∆*clpB* | ATCC 13032 derivative with in-frame deletion of the *clpB* gene (cg3079) | This study |
| ∆*malR* | ATCC 13032 derivative with in-frame deletion of the *malR* gene (cg3315) | ^43^ |
| *∆6C* | ATCC 13032 derivative with in-frame deletion of the *6C* gene (cgb_03605) | ^44^ |
| *∆6C*::*6C* | ATCC 13032 derivative with in-frame deletion of the *6C* gene (cgb_03605) with re-insertion of the *6C* gene (cgb_03605) | This study |
| MB001 | ATCC 13032 derivative with in-frame deletion of prophages CGP1 (cg1507-cg1524), CGP2 (cg1746-cg1752), and CGP3 (cg1890-cg2071) | ^25^ |
| MB001 ∆*ftsR* | MB001 derivative with in-frame deletion of the *ftsR* gene (cg1631) | ^19^ |
| MB001 ∆*ftsR*::P*gntK*-*ftsZ* | MB001 derivative with a chromosomal promoter exchange of the native *ftsZ*-promoter (cg2366) against the gluconate-inducible promoter of *gntK* (cg2732) and in-frame deletion of the *ftsR* gene (cg1631) | ^19^ |
| GRS51 | MB001 derivative with in-frame deletion of IS*cg12* and *rrnC*-cg3298 genes | ^45^ |
| GRS41_51 | MB001 derivative with in-frame deletion of IS*cg12*, *rrnC*-cg3298 and *ccrB*-cg2828 genes | ^45^ |
| MB001(DE3) | MB001 derivative with chromosomally encoded T7 gene 1 (cg1122-P*_lacI_*-*lacI*-P_lacUV5_–*lacZα*-gene 1-cg1121) | ^46^ |
| ChassisC1 | Genome-reduced strain | ^47^ |
| BOL-1 | ATCC 13032 derivative with in-frame deletions of *cat* (cg0310), *pqo* (cg2891), *pta-ackA* (cg3047-cg3048), and *ldhA* (cg3219) genes | ^48^ |
| BOL-2 | BOL-1 derivative with chromosomal integration into the *∆pta-ackA* (cg3047-cg3048) locus of the *pyc*^P458S^ gene (cg0791) from *C. glutamicum* DM1727 under the control of the *tuf* (cg0587) promoter | ^48^ |
| BOL-3 | BOL-2 derivative with chromosomal integration into the *∆pqo* (cg2891) locus of the *fdh* gene from *Mycobacterium vaccae* under the control of the *tuf* (cg0587)promoter from *C. glutamicum* | ^48^ |
| Cg43 | ATCC 13032 derivative with the following changes: P*trc*-*hisEG*(S143F/ΔC) P*trc*-*hisDCB* P*tuf*-*hisHAFI*  P36H-*hisN* | This study |
| DM1933 | ∆*pck pyc*(P458S) *hom*(V59A), 2 copies of *lysC*(T311I), 2 copies of *asd*, 2 copies of *dapA*, 2 copies of *dapB*, 2 copies of *ddh*, 2 copies of *lysA*, 2 copies of *lysE*, derived from WT *C. glutamicum* | ^49^ |
| DM1800 | *pyc*^P458S^*, lysC*^T311I^, derived from DM1727 | ^50^ |
| DM1800 ∆*prpC1* ∆*prpC2* | DM1800 derivative with in-frame deletion of *prpC1* (cg0798) and *prpC2* (cg0762) | This study |
| DM1800 ∆*prpC1* ∆*prpC2* ∆P*gltA*::P*dapA*(A14) | DM1800 derivative with in-frame deletion of *prpC1* (cg0798) and *prpC2* (cg0762) and exchange of the *gltA* (cg0949) promoter against the *dapA* (cg0762) promoter with a nucleotide exchange at position 14 | This study |
| DM1800 ∆*prpC1* ∆*prpC2* ∆P*gltA*::P*dapA*(A16) | DM1800 derivative with in-frame deletion of *prpC1* (cg0798) and *prpC2* (cg0762) and exchange of the *gltA* (cg0949) promoter against the *dapA* (cg0762) promoter with a nucleotide exchange at position 16 | This study |
| DM1800∆*prpC1*∆*prpC2*∆P*gltA*::P*dapA*(C17) | DM1800 derivative with in-frame deletion of *prpC1* (cg0798) and *prpC2* (cg0762) and exchange of the *gltA* (cg0949) promoter against the *dapA* (cg0762) promoter with a nucleotide exchange at position 17 | This study |
| DM1800 ∆*prpC1* ∆*prpC2* ∆P*gltA*::P*dapA*(L1) | DM1800 derivative with in-frame deletion of *prpC1* (cg0798) and *prpC2* (cg0762) and exchange of the *gltA* (cg0949) promoter against the *dapA* (cg0762) promoter with a nucleotide exchange at position L1 | This study |
| DM1800∆*lysEG* | DM1800 derivative with in-frame deletion of *lysEG* (cg1424-cg1425) | This study |
| DelAro^4^-*4cl^PCgc^*C7mu*fasO_BC_* | MB001(DE3) derivative with in-frame deletions of cg0344-347, cg0502, cg1226 and cg2625-2640, harboring a chromosomally encoded codon-optimized *4cl^Pc^* gene coding for 4-coumarate:CoA ligase from *Petroselinum crispum* (codon-optimized) under control of the T7 promoter (Δcg0344-0347::P_T7_-*4cl^Pc^*) and replacement of the native *gltA* promoter with the *dapA* (cg0762) promoter variant C7 (P*_gltA_*::P*_dapA_*-C7) and with a mutated FasR binding site upstream of *accBC* (cg0802) | ^51^ |
| JVO1 ∆*fkpA* | ATCC 13032 derivative *lysC*(T311I) *pyc*(P458S) ∆*prpC1* (cg0798) ∆*prpC2* (cg0762) ∆*fkpA* (cg0950) | ^52^ |
| MV-Leu20 | ATCC 13032 Δ*ltbR* (cg1486) Δ*leuA* (cg0303) derivative with chromosomal integration of *leuA*_B018 under control of the *tuf* (cg0587) promoter into the Δ*leuA* (cg0303) locus | ^53^ |
| MV-Leu20 Δ*ilvE* | MV-Leu20 derivative with in-frame deletion of *ilvE* (cg2418) | ^54^ |
| MV-Leu55 | MV-Leu20 Δ*ltbR*::P*_tuf_*-*leuA*_B018 derivative with chromosomally integrated mutations into *ilvN* (cg1436) coding for amino acid exchanges G20D, I21D, and I22F | ^53^ |
| MV-Leu55 Δ*ilvE* | MV-Leu55 derivative with in-frame deletion of *ilvE* (cg2418) | This study |
| ∆P*_atpB_*::P*_tuf_* | ATCC 13032 derivative with replacement of the native *atpB* (cg1362) promoter with the *tuf* (cg0587) promoter | This study |
| ∆P*_ctaD_*::P*_tuf_* ∆P*_ctaE_*::P*_tuf_* ∆P*_ctaC_*::P*_tuf_* | WT derivative with replacement of the native promoters of *ctaD* (cg2780), *ctaE* (cg2406) and *ctaC* (cg2409) with the *tuf* (cg0587) promoter | This study |
| ∆P*_nrdH_*::P*_tuf_* ∆P*_ctaE_*::P*_tuf_* ∆P*_ctaC_*::P*_tuf_* | WT derivative with replacement of the native *nrdH* (cg2789), *ctaE* (cg2406) and *ctaC* (cg2409) promoter with the *tuf* (cg0587) promoter | This study |
| **Plasmids** |  |  |
| pEKEx1 | Expression vector carrying *lacIq* and the *tac* promoter, Km^R^ | ^55^ |
| pEKEx2 | *Escherichia coli/C. glutamicum* shuttle vector; P*tacI*; *lacI*q; *oriV_C.g._* from pBL1*.*; *ori_E.c_.* ColE1 from pUC18, Km^R^ | ^56^ |
| pEKEx3 | *C. glutamicum*/*E. coli* shuttle vector for regulated gene expression, pBL1 *oriV_C.g_.*, pUC18 *oriV_E.c._*, P*tac*, *lacI*q, Spc^R^ | ^57^ |
| pJC1 | *E. coli/C. glutamicum* shuttle vector, Km^R^ | ^58^ |
| pAN6 | *C. glutamicum*/*E. coli* shuttle vector for regulated gene expression; derivative of pEKEx2 (*P*tac, *lacI* q, pBL1 *oriV_C.g._*, pUC18 *oriV_E.c_*_.),_ Km^R^ | ^26^ |
| pCLTON2 | *C. glutamicum* expression vector with the *B. subtilis* derived P*tet* promoter from pWH105 and the *tetR* gene under control of *C. glutamicum* P*gap* (cg1791) promoter from pJC1-p*gap*-*tetR*, Cm^R^ | ^59^ |
| pVWEx1 | P*tac*, *lacI*q; expression vector for *C. glutamicum*, Km^R^ | ^60^ |
| pMKEx2 | *C. glutamicum* vector for expression of target genes under control of the T7 promoter, based on pJC1 (P*lacI*, *lacI*, PT7, *lacO1*, N-term. Strep tagII, C-term. His tag, pHM1519 *oriC.g.*; pACYC177 *oriE.c.*), Km^R^ | ^26^ |
| pEKEx2-AmyE | pEKEx2 containing a gene encoding *Bacillus subtilis* AmyE with its authentic signal peptide and a  carboxyl-terminal His-tag, Km^R^ | ^61^ |
| pEKEx2-ΔSP-AmyE | pEKEx2 containing a gene encoding a signal peptide-less variant of *B. subtilis* AmyE with a carboxylterminal His-tag, Km^R^ | ^61^ |
| pEKEx2-AmyE(D217N) | pEKEx2 containing a gene encoding a signal peptide-less variant of *B. subtilis* AmyE (with amino acid substitution Asp -> Asn at position 217) with a carboxylterminal His-tag, Km^R^ | This study |
| pEKEx2-AmyH | pEKEx2 containing a gene encoding *Haloarcula hispanica* AmyH with its authentic signal peptide, Km^R^ | This study |
| pEKEx2-ΔSP-AmyH | pEKEx2 containing a gene encoding *H. hispanica* AmyH with its authentic signal peptide, Km^R^ | This study |
| pEKEx2-NprE-cutinase | pEKEx2 containing a gene encoding the mature part of the cutinase from *Fusarium solani pisi* fused to the NprE signal peptide from *B. subtilis*, Km^R^ | ^62^ |
| pEKEx2-GFP | pEKEx2 containing the *gfp* gene, Km^R^ | ^59^ |
| pEKEx2-*phoA* | pEKEx2 containing a gene encoding *E. coli* PhoA with its authentic signal peptide, Km^R^ | ^61^ |
| pEKEx2-PhoD-GFP | pEKEx2 containing the *gfp* gene fused to the PhoD signal peptide from *C. glutamicum*, Km^R^ | ^63^ |
| pEKEx2-TorA-GFP | pEKEx2 containing the *gfp* gene fused to the TorA signal peptide from *C. glutamicum*, Km^R^ | ^63^ |
| pEKEx2-TorA-cg2705 | pEKEx2 containing the cg2705 gene fused to the TorA signal peptide from *C. glutamicum*, Km^R^ | This study |
| pEKEx1-cg0955-GFP | pEKEx1 containing the *gfp* gene fused to the cg0955 signal peptide from *C. glutamicum*, Km^R^ | This study |
| pEKEx1-cg3287-GFP | pEKEx1 containing the *gfp* gene fused to the cg3287 signal peptide from *C. glutamicum*, Km^R^ | This study |
| pEKEx2-*Soxy* | pEKEx2 containing the Sorbitol/Xylitol oxidase gene of *Streptomyces coelicor* under the control of the *tac* promoter, Km^R^ | ^64^ |
| pEKEx2-TorA-*Soxy* | pEKEx2 containing the Sorbitol/Xylitol oxidase gene of *S. coelicor* fused to the signal peptide of the periplasmic Tat substrate trimethylamine N-oxide reductase from *E. coli* under the control of the *tac* promoter, Km^R^ | ^64^ |
| pEKEx2-iaTorA-*Soxy* | pEKEx2 containing the Sorbitol/Xylitol oxidase gene of *S. coelicor* fused to the inactivated signal peptide of the periplasmic Tat substrate trimethylamine N-oxide reductase from *E. coli* under the control of the *tac* promoter, Km^R^ | This study |
| pEKEx2-*cgtR1* | pEKEx2 containing the *cgtR1* gene (cg0330) from *C. glutamicum*, Km^R^ | This study |
| pJC1-*accBC* | pJC1 containing the *accBC* genes (cg0802) from *C. glutamicum*, Km^R^ | This study |
| pEKEx2-*cgtR10* | pEKEx2 containing the *cgtR10* gene (cg1084) from *C. glutamicum*, Km^R^ | This study |
| pEKEx2-*ihf* | pEKEx2 containing the *ihf* gene (cg1811) from *C. glutamicum*, Km^R^ | This study |
| pAN6-cg1914 | pAN6 containing the cg1914 gene from *C. glutamicum*, Km^R^ | This study |
| pAN6-*cgpS*-*strep* | pAN6 containing the *cgpS* gene (cg1966) from *C. glutamicum* without stop codon encoding a C-terminal Strep-tag fusion, Km^R^ | ^65^ |
| pAN6-*N-cgpS* | pAN6 containing the first 65 amino acids of the *cgpS* gene (cg1966) from *C. glutamicum*, Km^R^ | ^65^ |
| pAN6-cg1978 | pAN6 containing the cg1978 gene from *C. glutamicum*, Km^R^ | This study |
| pEKEx2-*cgtR6* | pEKEx2 containing the *cgtR6* gene (cg3061) from *C. glutamicum*, Km^R^ | This study |
| pEKEx2-*int2* | pEKEx2 containing the *int2* gene (cg2070) from *C. glutamicum*, Km^R^ | This study |
| pEKEx2-*cpdA* | pEKEx2 containing the *cpdA* gene (cg2761) from *C. glutamicum*, Km^R^ | ^39^ |
| pEKEx2-*malR* | pEKEx2 containing the *malR* gene (cg3315) from *C. glutamicum*, Km^R^ | ^43^ |
| pEKEx2-*hfq* | pEKEx2 containing a *hfq* gene from *E. coli*, Km^R^, different clones | This study |
| pAN6-*citH* | pAN6 containing the *citH* gene (cg0088) from *C. glutamicum*, Km^R^ | ^23^ |
| pJC1-*ilvBNCD* | pJC1 containing the *ilvBNCD* genes (cg1435-cg1437, cg1432) from *C. glutamicum*, Km^R^ | ^66^ |
| pAN6-cg0210 | pAN6 containing the cg0210 gene from *C. glutamicum*, Km^R^ | This work |
| pEKEx3-*esrR* | pEKEx3 containing the *esrR* gene (cg0709) from *C. glutamicum*, Km^R^ | ^9^ |
| pAN6-cg0764 | pAN6 containing the cg0764 gene *C. glutamicum*, Km^R^ | This work |
| pEKEx2-*mtrA* | pEKEx2 containing the *mtrA* gene (cg0862) from *C. glutamicum*, Km^R^ | ^67^ |
| pEKEx2-*mtrA*(Asp53Asn) | pEKEx2 containing the *mtrA*(D53N) gene (cg0862) from *C. glutamicum*, Km^R^ | ^67^ |
| pEKEx2-*cgtR2* | pEKEx2 derivative containing the *cgtR2* gene (cg0996) from *C. glutamicum*, Km^R^ | This work |
| pCLTON2-*cgtR2* | pCLTON2 derivative containing the *cgtR2* gene (cg0996) from *C. glutamicum*, Cm^R^ | This work |
| pAN6-*rosR* | pAN6 derivative containing the *rosR* gene (cg1324) from *C. glutamicum*, Km^R^ | ^17^ |
| pAN6-*ftsR* | pAN6 derivative containing the *ftsR* gene (cg1631) from *C. glutamicum*, Km^R^ | ^19^ |
| pVWEx1-cg1633 | pVWEx1 derivative containing the cg1633 gene from *C. glutamicum*, Km^R^ | This work |
| pVWEx1-*pup*-E64A | Derivative of pVWEx1-*pup* (cg1689) coding for a Pup protein with a Glu64Ala exchange, Km^R^ | ^21^ |
| pEKEx2-*acn* | pEKEx2 derivative containing the *acn* gene (cg1737) of *C. glutamicum* under the control of the *tac* promoter, Km^R^ | ^23^ |
| pEKEx2-*alpC* | pEKEx2 derivative containing the *alpC* gene (cg1890) of *C. glutamicum* under the control of the *tac* promoter, Km^R^ | This work |
| pEKEx2-*cgtR5* | pEKEx2 derivative containing the *cgtR5* gene (cg2947) of *C. glutamicum* under the control of the *tac* promoter, Km^R^ | This work |
| pJC1-*6C* | pJC1 derivative containing the *6C* gene of *C. glutamicum* under the control of the *tac* promoter, Km^R^ | ^44^ |
| pEKEx2-*cadA* | pEKEX2 derivative containing the *cadA* gene of *C. glutamicum* under the control of the *tac* promoter, Km^R^ | This study |
| pMKEx2-STS*AhCg*-4CL*PcCg* | pMKEx2 derivative containing codon-optimized genes coding for stilbene synthase from *Arachis hypogea* and 4-coumarate: CoA ligase from *Petroselinum crispum* under the control of the T7 promoter, Km^R^ | ^6^ |

### References

1 Abe, S., Takayama, K.-I. & Kinoshita, S. Taxonomical studies on glutamic acid-producing bacteria. *J Gen Appl Microbiol* **13**, 279-301 (1967).

2 Kinoshita, S., Udaka, S. & Shimono, M. Studies on the amino acid fermentation. Part 1. Production of L-glutamic acid by various microorganisms. *The Journal of General and Applied Microbiology* **3**, 193-205, doi:10.2323/jgam.3.193 (1957).

3 Brocker, M., Schaffer, S., Mack, C. & Bott, M. Citrate utilization by *Corynebacterium glutamicum* is controlled by the CitAB two-component system through positive regulation of the citrate transport genes *citH* and *tctCBA*. *J Bacteriol* **191**, 3869-3880, doi:10.1128/JB.00113-09 (2009).

4 Radmacher, E. *et al.* Linking central metabolism with increased pathway flux: L-valine accumulation by *Corynebacterium glutamicum*. *Appl Environ Microbiol* **68**, 2246-2250, doi:10.1128/aem.68.5.2246-2250.2002 (2002).

5 Klaffl, S., Brocker, M., Kalinowski, J., Eikmanns, B. J. & Bott, M. Complex regulation of the phosphoenolpyruvate carboxykinase gene *pck* and characterization of its GntR-type regulator IolR as a repressor of *myo*-inositol utilization genes in *Corynebacterium glutamicum*. *J Bacteriol* **195**, 4283-4296, doi:10.1128/JB.00265-13 (2013).

6 Kallscheuer, N. *et al.* Identification of the *phd* gene cluster responsible for phenylpropanoid utilization in *Corynebacterium glutamicum*. *Appl Microbiol Biotechnol* **100**, 1871-1881, doi:10.1007/s00253-015-7165-1 (2016).

7 Wolf, N. *et al.* Molecular Basis of Growth Inhibition by Acetate of an Adenylate Cyclase-Deficient Mutant of *Corynebacterium glutamicum*. *Front Microbiol* **11**, 87, doi:10.3389/fmicb.2020.00087 (2020).

8 Bussmann, M. *et al.* Transcriptional control of the succinate dehydrogenase operon *sdhCAB* of *Corynebacterium glutamicum* by the cAMP-dependent regulator GlxR and the LuxR-type regulator RamA. *J Biotechnol* **143**, 173-182, doi:10.1016/j.jbiotec.2009.06.025 (2009).

9 Kleine, B. *et al.* The three-component system EsrISR regulates a cell envelope stress response in *Corynebacterium glutamicum*. *Mol Microbiol* **106**, 719-741, doi:10.1111/mmi.13839 (2017).

10 Koçan, M. *et al.* Two-component systems of *Corynebacterium glutamicum*: deletion analysis and involvement of the PhoS-PhoR system in the phosphate starvation response. *J Bacteriol* **188**, 724-732, doi:10.1128/JB.188.2.724-732.2006 (2006).

11 Baumgart, M. & Frunzke, J. The manganese-responsive regulator MntR represses transcription of a predicted ZIP family metal ion transporter in *Corynebacterium glutamicum*. *FEMS Microbiol Lett* **362**, 1-10, doi:10.1093/femsle/fnu001 (2015).

12 Brocker, M. & Bott, M. Evidence for activator and repressor functions of the response regulator MtrA from *Corynebacterium glutamicum*. *FEMS Microbiol Lett* **264**, 205-212, doi:10.1111/j.1574-6968.2006.00456.x (2006).

13 Möker, N. *et al.* Deletion of the genes encoding the MtrA-MtrB two-component system of *Corynebacterium glutamicum* has a strong influence on cell morphology, antibiotics susceptibility and expression of genes involved in osmoprotection. *Mol Microbiol* **54**, 420-438, doi:10.1111/j.1365-2958.2004.04249.x (2004).

14 Engels, S., Schweitzer, J. E., Ludwig, C., Bott, M. & Schaffer, S. *clpC* and *clpP1P2* gene expression in *Corynebacterium glutamicum* is controlled by a regulatory network involving the transcriptional regulators ClgR and HspR as well as the ECF sigma factor sigmaH. *Mol Microbiol* **52**, 285-302, doi:10.1111/j.1365-2958.2003.03979.x (2004).

15 Wennerhold, J., Krug, A. & Bott, M. The AraC-type regulator RipA represses aconitase and other iron proteins from *Corynebacterium* under iron limitation and is itself repressed by DtxR. *J Biol Chem* **280**, 40500-40508, doi:10.1074/jbc.M508693200 (2005).

16 Kabus, A., Niebisch, A. & Bott, M. Role of cytochrome *bd* oxidase from *Corynebacterium glutamicum* in growth and lysine production. *Appl Environ Microbiol* **73**, 861-868, doi:10.1128/AEM.01818-06 (2007).

17 Bussmann, M., Baumgart, M. & Bott, M. RosR (Cg1324), a hydrogen peroxide-sensitive MarR-type transcriptional regulator of *Corynebacterium glutamicum*. *J Biol Chem* **285**, 29305-29318, doi:10.1074/jbc.M110.156372 (2010).

18 Koch-Koerfges, A., Kabus, A., Ochrombel, I., Marin, K. & Bott, M. Physiology and global gene expression of a *Corynebacterium glutamicum* ΔF_1_F_O_-ATP synthase mutant devoid of oxidative phosphorylation. *Biochim Biophys Acta* **1817**, 370-380, doi:10.1016/j.bbabio.2011.10.006 (2012).

19 Kraxner, K. J., Polen, T., Baumgart, M. & Bott, M. The conserved actinobacterial transcriptional regulator FtsR controls expression of *ftsZ* and further target genes and influences growth and cell division in *Corynebacterium glutamicum*. *BMC Microbiol* **19**, 179, doi:10.1186/s12866-019-1553-0 (2019).

20 Maeda, T., Koch-Koerfges, A. & Bott, M. Relevance of NADH Dehydrogenase and Alternative Two-Enzyme Systems for Growth of *Corynebacterium glutamicum* With Glucose, Lactate, and Acetate. *Front Bioeng Biotechnol* **8**, 621213, doi:10.3389/fbioe.2020.621213 (2020).

21 Küberl, A. *et al.* Pupylated proteins in *Corynebacterium glutamicum* revealed by MudPIT analysis. *Proteomics* **14**, 1531-1542, doi:10.1002/pmic.201300531 (2014).

22 Küberl, A., Polen, T. & Bott, M. The pupylation machinery is involved in iron homeostasis by targeting the iron storage protein ferritin. *Proc Natl Acad Sci U S A* **113**, 4806-4811, doi:10.1073/pnas.1514529113 (2016).

23 Baumgart, M., Mustafi, N., Krug, A. & Bott, M. Deletion of the aconitase gene in *Corynebacterium glutamicum* causes strong selection pressure for secondary mutations inactivating citrate synthase. *J Bacteriol* **193**, 6864-6873, doi:10.1128/JB.05465-11 (2011).

24 Donovan, C. *et al.* A prophage-encoded actin-like protein required for efficient viral DNA replication in bacteria. *Nucleic Acids Res* **43**, 5002-5016, doi:10.1093/nar/gkv374 (2015).

25 Baumgart, M. *et al.* Construction of a prophage-free variant of *Corynebacterium glutamicum* ATCC 13032 for use as a platform strain for basic research and industrial biotechnology. *Appl Environ Microbiol* **79**, 6006-6015, doi:10.1128/AEM.01634-13 (2013).

26 Frunzke, J., Engels, V., Hasenbein, S., Gätgens, C. & Bott, M. Co-ordinated regulation of gluconate catabolism and glucose uptake in *Corynebacterium glutamicum* by two functionally equivalent transcriptional regulators, GntR1 and GntR2. *Mol Microbiol* **67**, 305-322, doi:10.1111/j.1365-2958.2007.06020.x (2008).

27 Schreiner, M. E., Fiur, D., Holátko, J., Pátek, M. & Eikmanns, B. J. E1 enzyme of the pyruvate dehydrogenase complex in *Corynebacterium glutamicum*: molecular analysis of the gene and phylogenetic aspects. *J Bacteriol* **187**, 6005-6018, doi:10.1128/JB.187.17.6005-6018.2005 (2005).

28 Mahr, R. *et al.* Biosensor-driven adaptive laboratory evolution of l-valine production in *Corynebacterium glutamicum*. *Metab Eng* **32**, 184-194, doi:10.1016/j.ymben.2015.09.017 (2015).

29 Krüger, A. *et al.* Impact of CO_2_/HCO_3_^-^ availability on Anaplerotic Flux in Pyruvate Dehydrogenase Complex-Deficient *Corynebacterium glutamicum* Strains. *J Bacteriol* **201**, doi:10.1128/JB.00387-19 (2019).

30 Wennerhold, J. & Bott, M. The DtxR regulon of *Corynebacterium glutamicum*. *J Bacteriol* **188**, 2907-2918, doi:10.1128/JB.188.8.2907-2918.2006 (2006).

31 Helfrich, S. *et al.* Live cell imaging of SOS and prophage dynamics in isogenic bacterial populations. *Mol Microbiol* **98**, 636-650, doi:10.1111/mmi.13147 (2015).

32 Heyer, A. *et al.* The two-component system ChrSA is crucial for haem tolerance and interferes with HrrSA in haem-dependent gene regulation in *Corynebacterium glutamicum*. *Microbiology (Reading)* **158**, 3020-3031, doi:10.1099/mic.0.062638-0 (2012).

33 Georgi, T., Engels, V. & Wendisch, V. F. Regulation of L-lactate utilization by the FadR-type regulator LldR of *Corynebacterium glutamicum*. *J Bacteriol* **190**, 963-971, doi:10.1128/JB.01147-07 (2008).

34 Frunzke, J., Gätgens, C., Brocker, M. & Bott, M. Control of heme homeostasis in *Corynebacterium glutamicum* by the two-component system HrrSA. *J Bacteriol* **193**, 1212-1221, doi:10.1128/JB.01130-10 (2011).

35 Oertel, D., Schmitz, S. & Freudl, R. A TatABC-type Tat translocase is required for unimpaired aerobic growth of *Corynebacterium glutamicum* ATCC13032. *PLoS One* **10**, e0123413, doi:10.1371/journal.pone.0123413 (2015).

36 Davoudi, C. F., Ramp, P., Baumgart, M. & Bott, M. Identification of Surf1 as an assembly factor of the cytochrome *bc*_1_-*aa*_3_ supercomplex of Actinobacteria. *Biochim Biophys Acta Bioenerg* **1860**, 148033, doi:10.1016/j.bbabio.2019.06.005 (2019).

37 Morosov, X., Davoudi, C. F., Baumgart, M., Brocker, M. & Bott, M. The copper-deprivation stimulon of *Corynebacterium glutamicum* comprises proteins for biogenesis of the actinobacterial cytochrome *bc*_1_-*aa*_3_ supercomplex. *J Biol Chem* **293**, 15628-15640, doi:10.1074/jbc.RA118.004117 (2018).

38 Nickel, J., Irzik, K., van Ooyen, J. & Eggeling, L. The TetR-type transcriptional regulator FasR of *Corynebacterium glutamicum* controls genes of lipid synthesis during growth on acetate. *Mol Microbiol* **78**, 253-265, doi:10.1111/j.1365-2958.2010.07337.x (2010).

39 Schulte, J., Baumgart, M. & Bott, M. Identification of the cAMP phosphodiesterase CpdA as novel key player in cAMP-dependent regulation in *Corynebacterium glutamicum*. *Mol Microbiol* **103**, 534-552, doi:10.1111/mmi.13574 (2017).

40 Cramer, A., Gerstmeir, R., Schaffer, S., Bott, M. & Eikmanns, B. J. Identification of RamA, a novel LuxR-type transcriptional regulator of genes involved in acetate metabolism of *Corynebacterium glutamicum*. *J Bacteriol* **188**, 2554-2567, doi:10.1128/JB.188.7.2554-2567.2006 (2006).

41 Baumgart, M. *et al.* IpsA, a novel LacI-type regulator, is required for inositol-derived lipid formation in *Corynebacteria* and *Mycobacteria*. *BMC Biol* **11**, 122, doi:10.1186/1741-7007-11-122 (2013).

42 Schelder, S., Zaade, D., Litsanov, B., Bott, M. & Brocker, M. The two-component signal transduction system CopRS of *Corynebacterium glutamicum* is required for adaptation to copper-excess stress. *PLoS One* **6**, e22143, doi:10.1371/journal.pone.0022143 (2011).

43 Hünnefeld, M., Persicke, M., Kalinowski, J. & Frunzke, J. The MarR-Type Regulator MalR Is Involved in Stress-Responsive Cell Envelope Remodeling in *Corynebacterium glutamicum*. *Front Microbiol* **10**, 1039, doi:10.3389/fmicb.2019.01039 (2019).

44 Pahlke, J. *et al.* The small 6C RNA of *Corynebacterium glutamicum* is involved in the SOS response. *RNA Biol* **13**, 848-860, doi:10.1080/15476286.2016.1205776 (2016).

45 Unthan, S. *et al.* Chassis organism from *Corynebacterium glutamicum* -a top-down approach to identify and delete irrelevant gene clusters. *Biotechnol J* **10**, 290-301, doi:10.1002/biot.201400041 (2015).

46 Kortmann, M., Kuhl, V., Klaffl, S. & Bott, M. A chromosomally encoded T7 RNA polymerase-dependent gene expression system for *Corynebacterium glutamicum*: construction and comparative evaluation at the single-cell level. *Microb Biotechnol* **8**, 253-265, doi:10.1111/1751-7915.12236 (2015).

47 Baumgart, M. *et al.* *Corynebacterium glutamicum* Chassis C1*: Building and Testing a Novel Platform Host for Synthetic Biology and Industrial Biotechnology. *ACS Synth Biol* **7**, 132-144, doi:10.1021/acssynbio.7b00261 (2018).

48 Litsanov, B., Brocker, M. & Bott, M. Toward homosuccinate fermentation: metabolic engineering of *Corynebacterium glutamicum* for anaerobic production of succinate from glucose and formate. *Appl Environ Microbiol* **78**, 3325-3337, doi:10.1128/AEM.07790-11 (2012).

49 Blombach, B., Hans, S., Bathe, B. & Eikmanns, B. J. Acetohydroxyacid synthase, a novel target for improvement of L-lysine production by *Corynebacterium glutamicum*. *Appl Environ Microbiol* **75**, 419-427, doi:10.1128/AEM.01844-08 (2009).

50 Georgi, T., Rittmann, D. & Wendisch, V. F. Lysine and glutamate production by *Corynebacterium glutamicum* on glucose, fructose and sucrose: roles of malic enzyme and fructose-1,6-bisphosphatase. *Metab Eng* **7**, 291-301, doi:10.1016/j.ymben.2005.05.001 (2005).

51 Milke, L., Kallscheuer, N., Kappelmann, J. & Marienhagen, J. Tailoring *Corynebacterium glutamicum* towards increased malonyl-CoA availability for efficient synthesis of the plant pentaketide noreugenin. *Microb Cell Fact* **18**, 71, doi:10.1186/s12934-019-1117-x (2019).

52 Kallscheuer, N., Bott, M., van Ooyen, J. & Polen, T. Single-Domain Peptidyl-Prolyl *cis/trans* Isomerase FkpA from *Corynebacterium glutamicum* Improves the Biomass Yield at Increased Growth Temperatures. *Appl Environ Microbiol* **81**, 7839-7850, doi:10.1128/AEM.02113-15 (2015).

53 Vogt, M. *et al.* Pushing product formation to its limit: metabolic engineering of *Corynebacterium glutamicum* for L-leucine overproduction. *Metab Eng* **22**, 40-52, doi:10.1016/j.ymben.2013.12.001 (2014).

54 Vogt, M., Haas, S., Polen, T., van Ooyen, J. & Bott, M. Production of 2-ketoisocaproate with *Corynebacterium glutamicum* strains devoid of plasmids and heterologous genes. *Microb Biotechnol* **8**, 351-360, doi:10.1111/1751-7915.12237 (2015).

55 Eikmanns, B. J., Kleinertz, E., Liebl, W. & Sahm, H. A family of *Corynebacterium glutamicum*/*Escherichia coli* shuttle vectors for cloning, controlled gene expression, and promoter probing. *Gene* **102**, 93-98, doi:10.1016/0378-1119(91)90545-m (1991).

56 Eikmanns, B. J., Thum-Schmitz, N., Eggeling, L., Ludtke, K. U. & Sahm, H. Nucleotide sequence, expression and transcriptional analysis of the *Corynebacterium glutamicum* *gltA* gene encoding citrate synthase. *Microbiology (Reading)* **140 (Pt 8)**, 1817-1828, doi:10.1099/13500872-140-8-1817 (1994).

57 Hoffelder, M., Raasch, K., van Ooyen, J. & Eggeling, L. The E2 domain of OdhA of *Corynebacterium glutamicum* has succinyltransferase activity dependent on lipoyl residues of the acetyltransferase AceF. *J Bacteriol* **192**, 5203-5211, doi:10.1128/JB.00597-10 (2010).

58 J. Cremer, L. E. a. H. S. Cloning the *dapA dapB* cluster of the lysine-secreting bacterium *Corynebacterium glutamicum*. *Mol Gen Genet* **220**, 478-480 (1990).

59 Lausberg, F., Chattopadhyay, A. R., Heyer, A., Eggeling, L. & Freudl, R. A tetracycline inducible expression vector for *Corynebacterium glutamicum* allowing tightly regulable gene expression. *Plasmid* **68**, 142-147, doi:10.1016/j.plasmid.2012.05.001 (2012).

60 Peters-Wendisch, P. G. *et al.* Pyruvate carboxylase is a major bottleneck for glutamate and lysine production by *Corynebacterium glutamicum*. *J Mol Microbiol Biotechnol* **3**, 295-300 (2001).

61 Jurischka, S. *et al.* A secretion biosensor for monitoring Sec-dependent protein export in *Corynebacterium glutamicum*. *Microb Cell Fact* **19**, 11, doi:10.1186/s12934-019-1273-z (2020).

62 Hemmerich, J. *et al.* Combinatorial impact of Sec signal peptides from *Bacillus subtilis* and bioprocess conditions on heterologous cutinase secretion by *Corynebacterium glutamicum*. *Biotechnol Bioeng* **116**, 644-655, doi:10.1002/bit.26873 (2019).

63 Meissner, D., Vollstedt, A., van Dijl, J. M. & Freudl, R. Comparative analysis of twin-arginine (Tat)-dependent protein secretion of a heterologous model protein (GFP) in three different Gram-positive bacteria. *Appl Microbiol Biotechnol* **76**, 633-642, doi:10.1007/s00253-007-0934-8 (2007).

64 Scheele, S. *et al.* Secretory production of an FAD cofactor-containing cytosolic enzyme (sorbitol-xylitol oxidase from *Streptomyces coelicolor*) using the twin-arginine translocation (Tat) pathway of *Corynebacterium glutamicum*. *Microb Biotechnol* **6**, 202-206, doi:10.1111/1751-7915.12005 (2013).

65 Pfeifer, E. *et al.* Silencing of cryptic prophages in *Corynebacterium glutamicum*. *Nucleic Acids Res* **44**, 10117-10131, doi:10.1093/nar/gkw692 (2016).

66 Sahm, H. & Eggeling, L. D-Pantothenate synthesis in *Corynebacterium glutamicum* and use of *panBC* and genes encoding L-valine synthesis for D-pantothenate overproduction. *Appl Environ Microbiol* **65**, 1973-1979, doi:10.1128/AEM.65.5.1973-1979.1999 (1999).

67 Brocker, M., Mack, C. & Bott, M. Target genes, consensus binding site, and role of phosphorylation for the response regulator MtrA of *Corynebacterium glutamicum*. *J Bacteriol* **193**, 1237-1249, doi:10.1128/JB.01032-10 (2011).
