# Supplementary material for: A manually curated compendium of expression profiles for the microbial cell factory Corynebacterium glutamicum
Source: Sci Data. 2022 Oct 1;9:594. doi: 10.1038/s41597-022-01706-7 (PMC9526701; doi:10.1038/s41597-022-01706-7)
Supplement: Supplementary file 3 — Supplementary Table S3 [file 41597_2022_1706_MOESM3_ESM.docx]

**Supplementary Table S3.** List of sets with 0 (13), 500-1,000 (10) and more than 1,000 differentially regulated genes (one). The number of differentially regulated genes comprises all with at least a ≥2-fold lowered or ≥2-fold increased mRNA level in the respective set. Categories: (1) WT condition A *vs.* WT condition B. (2) Plasmid-based gene overexpression in parental strain *vs*. parental strain with empty vector control. (3) Deletion mutant *vs.* parental strain. (4) Gene silencing strain *vs.* parental strain. (5) Promoter exchange strain *vs.* parental strain. (6) Producer strain *vs.* WT or other producer strain.

| **Set ID** | **Set name** | **Category** | **# of differentially regulated genes** |
| --- | --- | --- | --- |
| 31 | WT (harvest in ethanol) vs. WT | 1 | 0 |
| 90 | WT pEKEx2-TorA-GFP (1 mM IPTG) vs. WT pEKEx2-TorA-GFP (w/o IPTG) | 2 | 0 |
| 91 | WT pEKEx2-TorA-cg2705 (1 mM IPTG, 1 hour) vs. WT pEKEx2 | 2 | 0 |
| 95 | WT pEKExL-cg0955-GFP (1 mM IPTG, 30 min) vs. WT pEKExL-cg0955-GFP (w/o IPTG, 30 min) | 2 | 0 |
| 96 | WT pEKExL-cg3287-GFP (1 mM IPTG) vs. WT pEKExL-cg3287-GFP (w/o IPTG) | 2 | 0 |
| 100 | WT pEKEx2-inactivated TorA-*Soxy* vs. WT pEKEx2 | 2 | 0 |
| 101 | WT pEKEx2-TorA-*Soxy* vs. WT pEKEx2-Soxy | 2 | 0 |
| 142 | ∆cg0764 (CGXII, 70 mM glucose) vs. WT (CGXII, 70 mM glucose) | 3 | 0 |
| 192 | WT pVWEx1-cg1633 (w/o MgSO_4_) vs. WT pVWEx1 (w/o MgSO_4_) | 2 | 0 |
| 211 | ∆*sigB* pEKEx2-AmyE (1 mM IPTG) vs. WT pEKEx2-AmyE (1 mM IPTG) | 2 | 0 |
| 233 | ∆*chrSA* (2.5 µM FeSO_4_) vs. WT (2.5 µM FeSO_4_) | 3 | 0 |
| 271 | ∆*6C* (CGXII, 4% glucose) vs. WT (CGXII, 4% glucose) | 3 | 0 |
| 276 | ∆*6C*::*6C* vs. WT | 3 | 0 |
|  |  |  |  |
| 39 | WT (3 mM Na_2_S) vs. WT | 1 | 516 |
| 249 | ∆*fasR* vs. WT | 3 | 528 |
| 292 | DM1800 ∆*lysEG* pEKEx2-*cadA* vs. DM1800 pEKEx3 | 6 | 532 |
| 172 | ∆*cydAB* ∆*qcrBAC* (BHI) vs. WT (BHI) | 3 | 566 |
| 252 | ∆*ctaD* vs. WT | 3 | 611 |
| 114 | WT pAN6-cg1978 vs. WT pAN6 | 2 | 707 |
| 42 | WT (100 µM bromoresveratol) vs. WT | 1 | 737 |
| 171 | ∆*cydAB* ∆*qcrBAC* (CGXII, 2 g/L peptone) vs. WT CGXII (2 g/l peptone) | 3 | 773 |
| 201 | ∆*acn* (4% glucose, 100 mM glutamat, 35 µM FeSO_4_) vs. WT (4% glucose, 100 mM glutamat, 35 µM FeSO_4_) | 3 | 799 |
| 124 | WT pAN6-*citH* (50 mM sodium citrate, 5 mM CaCl_2_) vs. WT pAN6-*citH* | 2 | 814 |
|  |  |  |  |
| 43 | WT (250 µM bromoresveratol) vs. WT | 1 | 1354 |
