# Supplementary material for: A manually curated compendium of expression profiles for the microbial cell factory Corynebacterium glutamicum
Source: Sci Data. 2022 Oct 1;9:594. doi: 10.1038/s41597-022-01706-7 (PMC9526701; doi:10.1038/s41597-022-01706-7)
Supplement: Supplementary file 4 — Supplementary Table S4 [file 41597_2022_1706_MOESM4_ESM.docx]

**Supplementary Table S4.** List of genes that are at least 30 times among the top 20 of regulated genes with at least a ≥2-fold lowered or ≥2-fold increased mRNA level among all sets. For a complete list of genes among the top 20 of regulated genes see Supplementary Table S5.

| **Locus tag** | **Gene**  **name** | **Annotation** | **Functional categorization** | **#^1^** |
| --- | --- | --- | --- | --- |
| cg0077 |  | hypothetical protein, VTC domain, conserved | Unknown function | 30 |
| cg0230 | *gltD* | glutamine 2-oxoglutarate aminotransferase, small subunit | Amino acid transport and metabolism | 31 |
| cg0470 | *htaB* | secreted heme transport-associated protein | Transport and metabolism of further metabolites | 44 |
| cg0760 | *prpB2* | 2-methylisocitrate lyase | Carbon source transport and metabolism | 39 |
| cg0762 | *prpC2* | 2-methylcitrate synthase | Carbon source transport and metabolism | 46 |
| cg0771 | *irp1* | putative siderophore ABC transporter, secreted substrate-binding lipoprotein | Inorganic ion transport, metabolism, and storage | 32 |
| cg0834 | *tusE* | ABC-type trehalose uptake system, solute-binding  lipoprotein | Carbon source transport and metabolism | 44 |
| cg0896 |  | putative membrane protein | Unknown function | 35 |
| cg0922 |  | putative secreted siderophore-binding lipoprotein | Inorganic ion transport, metabolism, and storage | 31 |
| cg0924 |  | putative ABC-type siderophore transporter, substrate-binding lipoprotein | Inorganic ion transport, metabolism, and storage | 52 |
| cg1930 |  | putative secreted hydrolase CGP3 region | Prophage genes | 33 |
| cg2071 | *int2* | putative phage integrase N-terminal fragment, CGP3 region | Prophage genes | 32 |
| cg2181 | *oppA* | ABC-type peptide transport system, secreted substrate-binding lipoprotein | Transport and metabolism of further metabolites | 30 |
| cg2183 | *oppC* | ABC-type peptide transport system, permease component | Transport and metabolism of further metabolites | 58 |
| cg2184 | *oppD* | ATPase component of peptide ABC-type transport system with  duplicated ATPase domains | Transport and metabolism of further metabolites | 50 |
| cg2560 | *aceA* | isocitrate lyase | Central carbon metabolism | 41 |
| cg2636 | *catA1* | catechol 1,2-dioxygenase | Carbon source transport and metabolism | 51 |
| cg2782 | *ftn* | ferritin | Inorganic ion transport, metabolism, and storage | 49 |
| cg2893 |  | putative MFS-type cadaverine transporter, multidrug efflux permease | Transport and metabolism of further metabolites | 36 |
| cg3048 | *pta* | phosphotransacetylase | Central carbon metabolism | 33 |
| cg3107 | *adhA* | Zn-dependent alcohol dehydrogenase | Carbon source transport and metabolism | 32 |
| cg3195 |  | putative flavin-containing monooxygenase | Transport and metabolism of further metabolites | 45 |
| cg3226 |  | L-lactate permease, operon with *lldD*, MFS-type | Carbon source transport and metabolism | 62 |
| cg3227 | *lldD* | menaquinone-dependent L-lactate dehydrogenase | Respiration and oxidative phosphorylation; carbon source transport and metabolism | 45 |
| cg3404 |  | putative ABC-type iron dicitrate transporter, substrate-binding lipoprotein | Inorganic ion transport, metabolism, and storage | 51 |

^1^Number of times a gene was among the top 20 of regulated genes with at least a ≥2-fold lowered or ≥2-fold increased mRNA level among all experiments.
